# Ending unjust HIV criminalization: leave no‐one behind

**DOI:** 10.1002/jia2.25681

**Published:** 2021-02-25

**Authors:** Edwin J Bernard, Chris Beyrer, Edwin Cameron, Michaela Clayton, Alexandra Volgina

**Affiliations:** ^1^ HIV Justice Network Amsterdam Netherlands; ^2^ Center for Public Health and Human Rights Johns Hopkins Bloomberg School of Public Health Baltimore MD USA; ^3^ Inspecting Judge of prisons and Chancellor Stellenbosch University Stellenbosch South Africa; ^4^ Health and Human Rights Consultant Windhoek Namibia; ^5^ Global Network of People Living with HIV Amsterdam Netherlands

**Keywords:** law and policy, structural drivers

In 2010, UNAIDS put forward their bold vision of the “Three Zeros,” to envisage a world in which there were zero new infections, zero AIDS deaths and zero discrimination [1]. One form of discrimination against people living with HIV that remains all too common a threat to their lives and wellbeing, as well as to the goal of ending the epidemic, is HIV criminalization. HIV criminalization describes the unjust application of criminal and similar laws to people living with HIV ostensibly based on their HIV status, either via HIV‐specific criminal statutes or general criminal or other laws. HIV criminalization laws are almost invariably exceedingly broad – either in their explicit wording or in the way they have been interpreted and applied. Many allow prosecution for acts that constitute no or very little risk by failing to recognize condom use or low viral load, or by criminalizing oral sex, or single acts of breastfeeding, biting, scratching or spitting. These laws – and their enforcement – are often based on myths, misconceptions and plain ignorance about HIV and its modes of transmission. As well as being a human rights issue of global concern, HIV criminalization is a barrier to universal access to HIV prevention, testing, treatment and care [2, 3].

UNAIDS is now calling on countries to adopt bold new targets to remove “societal and legal impediments to an enabling environment for HIV services”, which includes achieving a goal of fewer than 10% of countries with “punitive laws and policies” [4]. As of 2019, UNAIDS reports that 92 countries have either HIV‐specific criminal laws and/or have prosecuted individuals under general laws [4]. In 2020 alone, the HIV Justice Network (HJN) documented at least 90 unjust HIV criminalization cases across 25 countries [5]. Russia and the United States consistently prosecute the highest number of individuals, although a growing number of countries in Eastern Europe and Central Asia (EECA) and across sub‐Saharan Africa appear, inadvisedly, to be relying on HIV criminalization as a way of being seen to be doing something to address their rising HIV epidemics [5]. Migrants from higher HIV prevalence countries appear to be disproportionately prosecuted under HIV criminalization laws or policies in Canada, Europe and Australasia [6]. We are also seeing a frightening trend of prosecutions being initiated by those working in healthcare or public health without specific complaints. In some cases, police were notified of a person’s HIV diagnosis by health authorities, which then became a prompt to investigate the person’s relationship with their partner. For example, in Belarus and Uzbekistan there have been prosecutions against people living with HIV for consensual sex with HIV‐negative partners, even if their partner has consented and refused to have charges laid. Cases typically commence when healthcare providers hear that an HIV‐negative person is in a sexual relationship with a person living with HIV, or when a pregnancy is involved, or when a previously HIV‐negative partner tests HIV positive [6].

HIV criminalization is also a surrogate marker for state‐sponsored stigma and discrimination against marginalized groups of people at higher risk of HIV. HJN’s analysis of recent cases suggests that the likelihood of prosecution is exacerbated by intersectional discrimination or criminalization on the basis of ethnicity, sex, gender identity, immigration status, sex work, sexuality and/or substance use. Increasingly, cases in sub‐Saharan Africa and in EECA illustrate what we have long‐feared: that women in these regions are more likely to be prosecuted, since they are often the first in a relationship to know their status because of routine HIV testing during pregnancy, and are less likely to be able to safely disclose their HIV‐positive status to their partner as a result of inequality in power relations, economic dependency and high levels of gender‐based violence within relationships [6]. Women with HIV also face the possibility of being prevented from becoming pregnant, and/or of being prosecuted for passing HIV on to their child during pregnancy, birth or breastfeeding, further constricting their reproductive choices and rights. In 2020, women living with HIV were accused in 25% of all reported HIV criminalization cases; three of these cases associated with breastfeeding [5].

In the United States, more than 50% of those accused in reported HIV criminalization cases in 2020 were people of colour, a greater proportion than people of colour estimated to be living with HIV in the US [5, 7]. Cases in the United States also appear to disproportionately impact people already in the purview of the criminal justice system, such as prisoners, and people living in poverty, including homeless people, with a high number of cases related to supposed “HIV exposure” through biting or spitting during arrest or while incarcerated. The United States has not only been a world leader in HIV criminalization laws and prosecutions, it was previously also a leading exporter – either directly via financial and policy support via USAID [8], or by example given it was the first country to enact HIV‐specific criminal laws – in 1987 – and because media reports of high‐profile cases continue to be reported around the globe. However, given the US Centers for Disease Control and Prevention’s recent call for "the reforming, rescinding, and revising the application of [HIV criminalization] laws for the sake of people with HIV and for the public’s health," [9] and the new US administration’s move towards evidence‐based policymaking with global impact, such as rescinding the Protecting Life in Global Health Assistance Policy (PLGHA, previously known as the Mexico City Policy) [10], we hope that this now leads to an accelerated move to evidence‐based laws on HIV criminalization across the US, and globally.

Despite global recommendations to ensure HIV criminalization laws at least reflect current scientific evidence [2, 3] – *if they should exist at all* – relatively few countries have repealed or modernized their laws, although efforts are currently underway across the globe [6]. In July 2018, the Journal of the International AIDS Society published the *Expert consensus statement on the science of HIV in the context of criminal law* authored by 20 of the world’s leading HIV scientists, and endorsed by the International AIDS Society, UNAIDS, the International Association of Providers of AIDS Care, and more than 70 other influential HIV scientists and clinicians [11]. The Statement describes the latest evidence on HIV transmission risks, treatment effectiveness and forensics, so that HIV‐related science may be better understood in criminal law contexts. An interim scoping report found that the consensus statement has been instrumental in helping the criminal justice system, and law and policymakers, understand HIV‐related science, helping to shape advocacy for law and policy reform and improving legal and judicial practice [12].

The consensus statement makes it clear that people living with HIV, regardless of their access to treatment or prevention commodities, pose much less of a risk to their sexual partners on a per‐act basis than is widely believed by the criminal justice system. Thus, it is imperative that we strive not only to reform or modernize these laws, but to remove them from the statute books entirely, otherwise those people who are most marginalized – the poor, the already criminalized, those with mental health or problematic substance use who are unable to achieve viral suppression or to ensure consistent correct condom use – will continue to be unjustly prosecuted. If all of us involved in the HIV response are serious about ending HIV as a global pandemic, then we must make sure that everyone feels welcome when they take an HIV test or receives HIV treatment, and can talk openly and confidently with their sexual partners or healthcare workers about their lives without fearing judgment, or worse, being reported to the police.

On this Zero Discrimination Day, we celebrate the courage and commitment of the growing global community of advocates, human rights defenders and others around the world who are challenging laws, policies and practices that inappropriately and unjustly criminalize people living with HIV. Ending HIV criminalization is the responsibility of us all. That is why it is important that we all understand how to ensure justice for *all* people living with HIV, not just those who have access to treatment and are fortunate enough to be undetectable, so that we can finally end these outrageously unjust laws, policies and practices against people living with HIV in all of their diversity.

## COMPETING INTERESTS

The authors have declared no conflict of interest.

## AUTHORS’ CONTRIBUTIONS

The authors collaboratively conceived the content of the paper. EJB wrote the first draft. CB, EC, MC and AV edited the manuscript. EJB edited it into its final form.
